# Supplementary material for: Does frequency of supportive supervisory visits influence health service delivery?—Dose and response study
Source: PLoS One. 2020 Jun 16;15(6):e0234819. doi: 10.1371/journal.pone.0234819 (PMC7297318; doi:10.1371/journal.pone.0234819)
Supplement: S1 Data — (PDF) [file pone.0234819.s002.pdf]

FOLLOW-UP CHECKLIST: HEALTH CENTER (HC)					
Name of Health Center (HC)		Visitors			
		Name	Organization	Responsibility	Signature
Region					
Cluster					
Distance to facility					
Zone					
Woreda					
Woreda performance tier	1- High performing<br>2- Medium performing<br>3- Low performing				
Date (G.C)	DD / MM / YYYY				
Person in-charge during the visit in the HC					
GPS Coordinate (in six digits) at the main gate		Longitude			
		Latitude			

Instruction for responses: Score **1** for **Yes** responses, **2** for **No** responses, **NA** for **Not Applicable** and write the **value/#** for numeric questions.

SN	Questions	Yes=1, No=2, #, NA	Remarks
I	***Basic information***		
1.1	Catchment population of the HC		
1.2	**Total number** of Health Posts under the HC		
1.3	**Number** of outreach sites (EPI, FP, etc.)		
1.4	**Number** of technical staff in the HC		
1.5	**Number** of supportive staff in the HC		
1.6	Availability of water		
1.7	Availability of electricity		
1.8	Facility distance from woreda distance		
1.9	Facility head experience, in years		
II	***Access***		
2.1	Provide all exempted health services free of charge		
2.2	Provide health care services to CBHI beneficiaries		
2.3	Access to roads		
2.4	Has at least one ambulance		
III	***Patient centered care***		
3.1	Trained staff use chart booklets while providing services		
3.2	Delivery partograph is used correctly		
3.3	Under-five children classified correctly		
3.4	Under-five children treated correctly		
IV	***Service organization and management***		
4.1	Reviewed and reported EHCRIG chapters in the most recent quarter		

SN	Questions	Yes=1, No=2, #, NA	Remarks
4.2	Followed IPLS standards to ensure uninterrupted supply chain		
4.3	Used HMIS data for planning and decision making		
4.4	Used LQAS for data accuracy check		
4.5	HC Director trained on Leadership, Management and Governance (LMG)		
4.6	Established case review/audit system for maternal and newborn death		
4.7	Have an EPI defaulter tracing mechanism		
4.8	Established a QI team and assigned a focal person for QA/QI		
V	***Service availability***		
5.1	All expected FP methods are available in all days in the past one month		
5.2	PPFP service is available in delivery room		
5.3	Provided all BEmONC signal functions		
5.4	Provided women friendly delivery services		
5.5	Provided ferrous sulfate for pregnant women during ANC		
5.6	Functional maternity waiting room/home		
5.7	ANC clients tested for syphilis		
5.8	Mothers received Uterotonics in the third stage of labor or immediately after birth		
5.9	Newborns received newborn care		
5.10	Newborns with neonatal sepsis received treatment		
5.11	Asphyxiated newborns resuscitated		
VI	***Population health management***		
6.1	Exercise community feedback collecting mechanisms/town hall meetings		
6.2	Have a social behavior change communication plan		
6.3	Work together with kebele administration		
VII	***Major challenges and actions taken by the visited HC***		

At the end of the visit

1. Hold brief meeting with the HC personnel and reflect on key findings
2. Identify things that need to be followed by HC, WorHO, ZHD and RHB.
3. Develop action plan
